# Canine infection with *Borrelia burgdorferi, Dirofilaria immitis*, *Anaplasma* spp. and *Ehrlichia* spp. in Canada, 2013–2014

**DOI:** 10.1186/s13071-017-2184-7

**Published:** 2017-05-19

**Authors:** Brian H. Herrin, Andrew S. Peregrine, Jonas Goring, Melissa J. Beall, Susan E. Little

**Affiliations:** 1 Department of Veterinary Pathobiology, Center for Veterinary Health Sciences, Oklahoma State University, Stillwater, OK USA; 2 Department of Pathobiology, Ontario Veterinary College, University of Guelph, Guelph, ON Canada; 3 IDEXX Laboratories, Inc, Westbrook, ME USA

**Keywords:** *Borrelia burgdorferi*, *Dirofilaria immitis*, *Ehrlichia*, *Anaplasma*, Canada, Canine

## Abstract

**Background:**

Canine test results generated by veterinarians throughout Canada from 2013–2014 were evaluated to assess the geographical distribution of canine infection with *Borrelia burgdorferi*, *Dirofilaria immitis*, *Ehrlichia* spp., and *Anaplasma* spp.

**Methods:**

The percent positive test results of 115,636 SNAP® 4Dx® Plus tests from dogs tested were collated by province and municipality to determine the distribution of these vector-borne infections in Canada.

**Results:**

A total of 2,844/115,636 (2.5%) dogs tested positive for antibody to *B. burgdorferi*. In contrast, positive test results for *D. immitis* antigen and antibodies to *Ehrlichia* spp. and *Anaplasma* spp. were low, with less than 0.5% of dogs testing positive for any one of these three agents nationwide. Provincial seroprevalence for antibodies to *B. burgdorferi* ranged from 0.5% (Saskatchewan)–15.7% (Nova Scotia); the areas of highest percent positive test results were in proximity to regions in the USA considered endemic for Lyme borreliosis, including Nova Scotia (15.7%) and Eastern Ontario (5.1%). These high endemic foci, which had significantly higher percent positive test results than the rest of the nation (*P* < 0.0001), were surrounded by areas of moderate to low seroprevalence in New Brunswick (3.7%), Quebec (2.8%), and the rest of Ontario (0.9%), as well as northward and westward through Manitoba (2.4%) and Saskatchewan (0.5%). Insufficient results were available from the westernmost provinces, including Alberta and British Columbia, to allow analysis.

**Conclusion:**

Increased surveillance of these vector-borne disease agents, especially *B. burgdorferi*, is important as climate, vector range, and habitat continues to change throughout Canada. Using dogs as sentinels for these pathogens can aid in recognition of the public and veterinary health threat that each pose.

## Background

Vector-borne diseases are an emerging concern for veterinarians and physicians in much of Canada. The prevalence of vector-borne infections, including Lyme borreliosis (LB), is increasing, apparently due to changing environmental and climatic conditions [1–3]. Lyme borreliosis, heartworm, anaplasmosis, and ehrlichiosis are four common vector-borne diseases that are regularly diagnosed in dogs in the USA [4]. Determining the range and prevalence of the agents that cause these diseases throughout Canada may enhance awareness of their importance, encouraging preventive measures and leading to prompt, accurate diagnosis and appropriate treatment.

Canine LB in North America is caused by infection with the spirochete *Borrelia burgdorferi* (*sensu stricto*); other LB agents reported from people have not been identified in dogs. Disease in dogs is characterised by fever, lethargy, anorexia, and lymphadenopathy, but can progress to more severe manifestations such as arthritis and glomerulonephritis [5]. Transmission to humans and dogs is by *Ixodes* sp. ticks; *I. scapularis* is the vector for the eastern half of Canada and *I. pacificus* the most important vector in British Columbia [6]. Ticks harbouring *B. burgdorferi* have been identified throughout central and eastern Canada, including parts of Manitoba, Ontario, Quebec, Nova Scotia, and New Brunswick [7]. LB-endemic areas of Canada are defined as locations where all three life stages of the tick (larva, nymph, adult) have been collected for two consecutive years and *B. burgdorferi* infection has been confirmed in ticks or vertebrate hosts [8]. LB is the most commonly reported vector-borne disease in people in the USA [9]; approximately 25,000 cases are reported each year in the USA, while in Canada, approximately 900 new cases were reported in 2015, growing from only 140 cases in 2009 [10, 11]. This higher risk of infection in the US is also seen in pet dogs. Between 2012 and 2014, 7.2% of dogs tested had antibodies to *B. burgdorferi* in the USA [12]. In contrast, only 0.7% and 2.1% of dogs were reported to test positive in Canada in 2008–2010, respectively [13, 14].

*Dirofilaria immitis*, the causative agent of canine heartworm disease, is considered the most important helminth infection of dogs in the United States [4]. Mosquito vectors acquire *D. immitis* microfilariae when feeding on infected dogs and transmit the third-stage larvae, which then migrate and develop within dogs [15, 16]. The presence of adult heartworms in the pulmonary vasculature is a potential source of significant pathology [17–19]. Heartworm infection has been reported in dogs in Canada since 1977, but the prevalence has remained relatively low at around 0.2% [13, 20]. Because heartworm has historically been relatively uncommon in the region, most Canadian veterinary parasitologists recommend a seasonal preventive strategy. In addition, yearly testing is recommended for patients in high-risk groups, including dogs who travel to endemic areas or those not receiving any preventive, or those on a preventive with poor compliance [21]. Interestingly, over 77% of dogs that tested positive for infection with *D. immitis* in one report had no travel history outside the region, supporting autochthonous infection, albeit at a low level [13].

The rickettsial agents *Anaplasma phagocytophilum*, *A. platys*, *Ehrlichia canis*, and *E. ewingii* are all tick-borne bacterial pathogens infecting leukocytes or platelets of their host [22]. These agents induce similar clinical signs and laboratory findings ranging from fever, anorexia, myalgia, and thrombocytopenia to severe manifestations such as epistaxis and death [22].

*Anaplasma phagocytophilum* is transmitted through the bite of an *Ixodes* spp. tick*,* and is the causative agent of human granulocytic anaplasmosis (HGA) [23]. Previous canine serologic surveys in Canada have reported that the prevalence of dogs with antibodies to *A. phagocytophilum* is rising, with no dogs testing positive in 2006 but a prevalence ranging between 0.2–1.1% just five years later [13, 14, 24]. *Anaplasma platys*, causative agent of canine cyclic thrombocytopenia, is transmitted by *Rhipicephalus sanguineus* and infects platelets of dogs [25, 26]. In a previous study, 1.8% of dogs tested in Canada were reported to have antibodies to *A. platys* [14].

*Ehrlichia canis* is the causative agent of canine monocytic ehrlichiosis, and is also transmitted by *R. sanguineus*; infection causes anaemia, thrombocytopenia, and, in severe cases, potentially fatal bleeding diathesis [27, 28]. *Ehrlichia ewingii* is the causative agent of canine granulocytic ehrlichiosis and is transmitted by *Amblyomma americanum.* The range of *A. americanum* has dramatically expanded northward and eastward in recent decades [29]. While *A. americanum* populations are not yet considered established in Canada, the tick is occasionally reported from domestic dogs in Ontario with no travel history out of the region (Peregrine unpublished). Of the two, only *E. canis* has been reported in Canada previously, with 3.2% of dogs tested having antibodies to the pathogen, while 0/285 dogs tested positive for *Ehrlichia chaffeensis* or *E. ewingii* [14].

Evidence of past or current infection with all of these pathogens can be identified with assays commonly used for annual heartworm testing and as a screening tool for tick-borne infections, and the composite results can be evaluated on both a local and national level. For example, by reviewing the changing prevalence of antibody-positive dogs over time, previously undocumented areas of expansion of LB were detected [4, 12]. The present paper seeks to build on previous publications [13, 14], potentially identifying areas of recent expansion of LB as well as monitoring the overall distribution of these vector-borne infections in Canada.

## Methods

### Source of data

The data collected were obtained from the SNAP® 4Dx® Plus Test kit (IDEXX Laboratories, Inc., Westbrook, Maine, USA), an in-clinic ELISA for the simultaneous detection of canine antibodies to *B. burgdorferi, A. phagocytophilum, A. platys*, *E. canis,* and *E. ewingii,* and antigen of *D. immitis.* The results were generated from January 2013 through December 2014 by veterinarians testing patients in-clinic and recording the data manually or by IDEXX SnapShot Dx® instrumentation. For privacy, results were provided with no patient or owner identification; therefore, travel history, confirmatory diagnostics, and clinical outcome for each result is not known.

### *Borrelia burgdorferi* assay

The analyte utilised for the *B. burgdorferi* assay is the C_6_ peptide, which detects antibodies to a surface lipoprotein of *B. burgdorferi* (*sensu stricto*). The sensitivity and specificity of the analyte are reported in the package insert to be 94.1 and 96.2% (IDEXX Laboratories, Inc., Westbrook, ME, USA), respectively, but published studies with different populations report different values. For example, in comparison to a two-tiered, gold standard diagnostic process utilising immunofluorescence (IFA) and Western blot (WB), the test sensitivity was 94.4% [30, 31], and the test specificity has been reported to be 99.5% when used on field samples from dogs [30, 32]. The C_6_ analyte has also been shown to not cross-react with other *Borrelia* spp. found in the USA or react to antibodies produced through vaccination [30].

### Heartworm assay

The assay utilised detects *D. immitis* antigen primarily produced from the uterus of female heartworms. The sensitivity and specificity reported for the heartworm portion of the assay are 99.0 and 99.3%, respectively (IDEXX Laboratories, Inc.). Other studies have reported the sensitivity of this analyte as 84%, but that value varies with the intensity of infection, with a sensitivity of 64% when only one adult, female heartworm is present and 98% when 4 or more adult heartworms are present [31, 33].

### *Anaplasma* assay

A single analyte used that detect antibodies to a peptide from the MSP2/p44 major surface protein of two distinct *Anaplasma* spp.: *A. phagocytophilum* and *A. platys*. Detection of *A. platys* was added after recognising significant cross-reactivity (SNAP® 4Dx® Test kit insert, IDEXX Laboratories, Inc.). The reported sensitivity and specificity of the test are 90.3% and 94.3%, respectively (IDEXX Laboratories, Inc.). The sensitivity of the assay is 99.1% for *A. phagocytophilum* and 89.1% for *A. platys* when compared to IFA, while the specificities are reported as 100 and 99.8%, respectively, although sensitivity and specificity against field samples may vary [34, 35].

### *Ehrlichia* assay

Analytes were used that detect antibodies to the p30 and p30-1 proteins of *E. canis* and the p28 protein of *E. ewingii*. The reported sensitivity and specificity of this assay is 97.1 and 95.3%, respectively (IDEXX Laboratories, Inc.). In other studies, when compared to IFA or WB, the sensitivity was 95.7% for *E. canis* and 96.5% for *E. ewingii* [35, 36]. The test specificity for *E. canis* has been shown to be 100% [30, 37], while specificity for the detection of antibodies to *E. ewingii* is 93.9% [35]. Infection with other *Ehrlichia* spp. may induce cross-reactive antibodies leading to a positive test result on the *Ehrlichia* spp. analyte [30, 38].

### Data and statistical analysis

Data were collated by a three-digit postal code of the veterinary practice where the test was performed and then assembled into municipalities or major metropolitan areas and provinces. Only municipalities reporting more than 30 test results were included in the study. Percent positive test results were calculated by dividing the number of dogs with a positive test result by the total number of test results reported for each agent of interest. For all samples, 95% confidence intervals were calculated using the modified Wald method (GraphPad Software, La Jolla, CA, USA). Maps were assembled using the Canada base map and the Hatch Map function on MapViewer 7 (Golden Software, Golden, CO, USA), which provides base maps of Canada, and then modified using the hatch map function in the software.

Differences in reported frequency of positive test results between municipalities and provinces were evaluated using a Chi-square test in StatPlus (Windows 7, Redmond, WA; AnalystSoft, Alexandria, VA, USA) with significance assigned at *P* < 0.0001 as previously described [4].

## Results

Test results were available from a total of 225 practices in 2013 and 198 practices in 2014, representing 115,636 data points from 84 different municipalities across Canada. Ontario reported the highest number of test results (77,143) followed by Quebec (23,701), Manitoba (12,765), New Brunswick (1,631), Nova Scotia (210), and Saskatchewan (186). All other provinces and territories had fewer than 30 test results reported in a single municipality.

### *Borrelia burgdorferi*

**Fig. 1:**
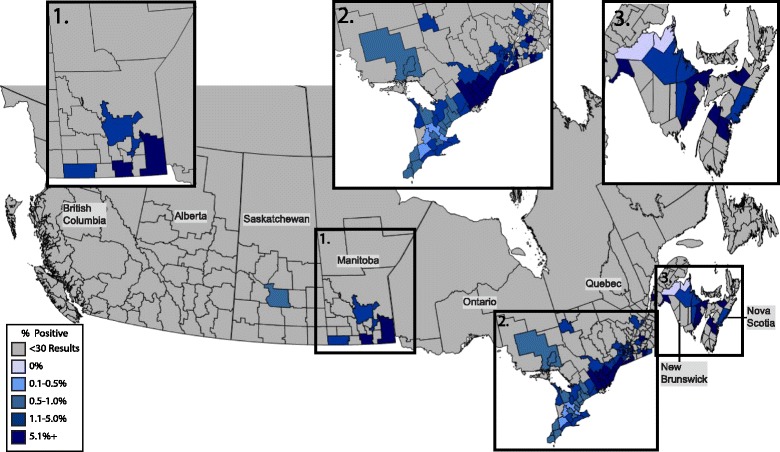
Percent positive antibody tests to *Borrelia burgdorferi* in dogs by municipality. Evidence of antibody to *Borrelia burdorferi* in dogs by municipality throughout Canada, 2013–2014, grouped according to percent positive tests

The prevalence of antibody positive dogs nationwide was 2.5% (2,844/115,636) with provincial prevalence ranging from 0.5–15.7% (Table 1). Over half (44/84) of the municipalities reported 2% or greater positive test results, while 7 reported less than 0.5% positive test results (Fig. 1). Positive test results for antibodies to *B. burgdorferi* were most common in Nova Scotia, with 15.7% of samples from this province testing positive, which was higher than the national average (*χ* ^2^ = 148.591, *P* < 0.0001). Other provinces had percent positive test results higher than the national average, including New Brunswick (3.7%; *χ* ^2^ = 9.743, *P* = 0.0018), and Quebec (2.8%; *χ* ^2^ = 15.456, *P* < 0.0001) (Fig. 1). Ontario had a lower overall seroprevalence than the national average (2.3%; *χ* ^2^ = 22.140, *P* < 0.0001), but in a cluster of 11 municipalities in eastern Ontario more than 5.1% (1,335/26,081; *χ* ^2^ = 991.266, *P* < 0.0001) of dogs tested positive.

**Table 1:** Vector-borne infections in dogs in Canada, 2013–2014. Percent positive test results (95% confidence intervals, CI), and total number positive by province for dogs tested from 2013–2014 for antigen of *Dirofilaria immitis* and antibody to *Borrelia burgdorferi*, *Ehrlichia* spp. and *Anaplasma *spp.

Province (Number of tests)	*Borrelia burgdorferi* % (95% CI) [No. positive]	*Dirofilaria immitis* % (95% CI) [No. positive]	*Anaplasma* spp. % (95% CI) [No. positive]	*Ehrlichia* spp. % (95% CI) [No. positive]
Manitoba (*n* = 12,765)	2.4 (2.1–2.7) [303]	0.20 (0.12–0.28) [26]	0.86% (0.70–1.0) [110]	0.24% (0.16–0.32) [31]
New Brunswick (*n* = 1,631)	3.7 (2.9–4.7) [60]	0.12 (0.01–0.48) [2]	0.43 (0.19–0.90) [1]	0.12 (0.01–0.48) [2]
Nova Scotia (*n* = 210)	15.7 (11.4–21.3) [33]	0.48 (0.01–2.9) [1]	0.95 (0.04–3.6) [2]	0 (0–2.2) [0]
Ontario (*n* = 77,143)	2.3 (2.2–2.4) [1,780]	0.50 (0.45–0.55) [385]	0.22 (0.19–0.25) [166]	0.19 (0.16–0.22) [146]
Quebec (*n* = 23,701)	2.8 (2.6–3.0) [667]	0.30 (0.23–0.37) [71]	0.19 (0.13–0.25) [46]	0.16 (0.11–0.21) [37]
Saskatchewan (*n* = 186)	0.54 (0.01–3.3) [1]	0 (0–2.4) [0]	0 (0–2.4) [0]	1.6 (0.33–4.9) [3]
National (*n* = 115,636)	2.5 (2.4–2.6) [1,844]	0.42 (0.38–0.46) [485]	0.29 (0.26–0.32) [331]	0.19 (0.16–0.22) [219]

### *Dirofilaria immitis*

**Fig. 2:**
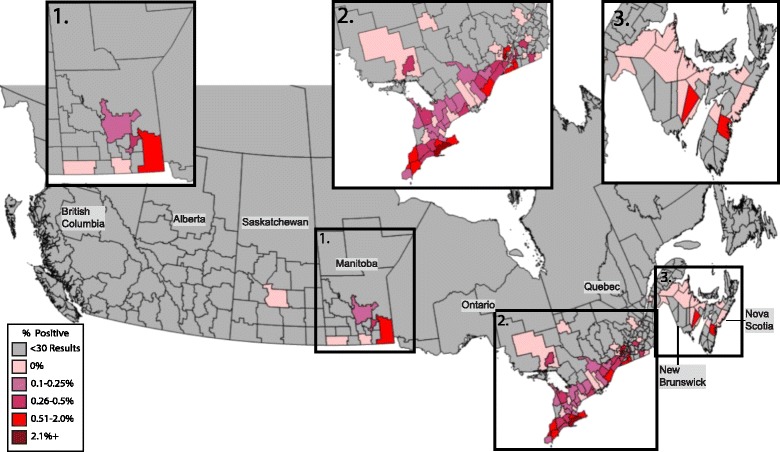
Percent positive antigen tests of *Dirofilaria immitis* in dogs by municipality. Evidence of antigen of *Dirofilaria immitis* in dogs by municipality throughout Canada, 2013–2014, grouped according to percent positive tests

Nationwide, 0.42% (485/115,636) of dogs tested positive for heartworm antigen, and no province had percent positive test results greater than 0.5% (0–0.5%) (Table 1). Ontario had the highest percent positive tests (0.50%). Two municipalities had percent positive test results higher than 2%: Mirabel, just west of Montreal, Quebec (5.0%; 2/40; 95% CI: 0.50–17.4%) and Caledonia, in southern Ontario near Toronto (4.1%; 207/5,111; 95% CI: 3.5–4.6%) (Fig. 2). Both municipalities had a higher prevalence than the national average and the rest of the respective province (*χ* ^2^ = 10.627, *P* = 0.0011; *χ* ^2^ = 1678.59, *P* < 0.0011).

### *Anaplasma* spp

**Fig. 3:**
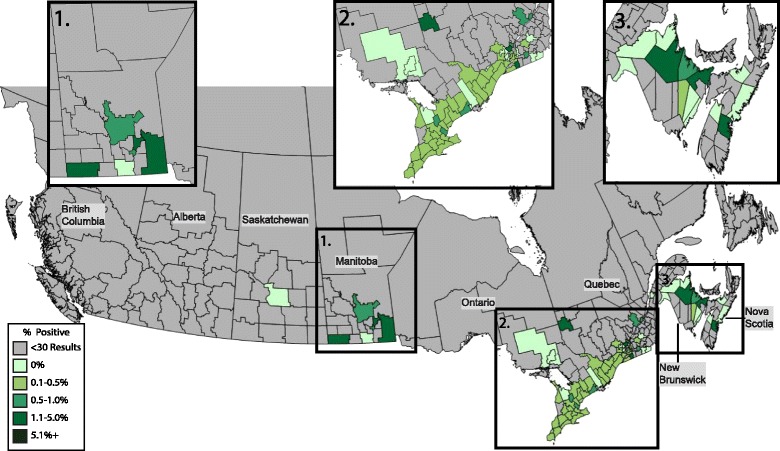
Percent positive antibody tests to *Anaplasma* spp. in dogs by municipality. Evidence of antibody to *Anaplasma* spp. in dogs by municipality throughout Canada, 2013–2014, grouped according to percent positive tests

Antibody to *Anaplasma* spp. was detected in 0.29% (331/115,636) of dogs, with a provincial seroprevalence ranging from 0.0–0.95% (Table 1). Nova Scotia and Manitoba were the only provinces that had a higher prevalence than the national average with 0.95%, and 0.86% of all tests reported positive, respectively; the total number of positive tests in municipalities within these provinces that had a seroprevalence above 1.0% ranged between 2 and 12 positive tests (Fig. 3). Percent positive test results in Ontario were significantly lower than the national average at 0.22% (*χ* ^2^ = 40.252, *P* < 0.0001); no municipalities in Ontario had percent positive test results over 1.0%.

### *Ehrlichia* spp

**Fig. 4:**
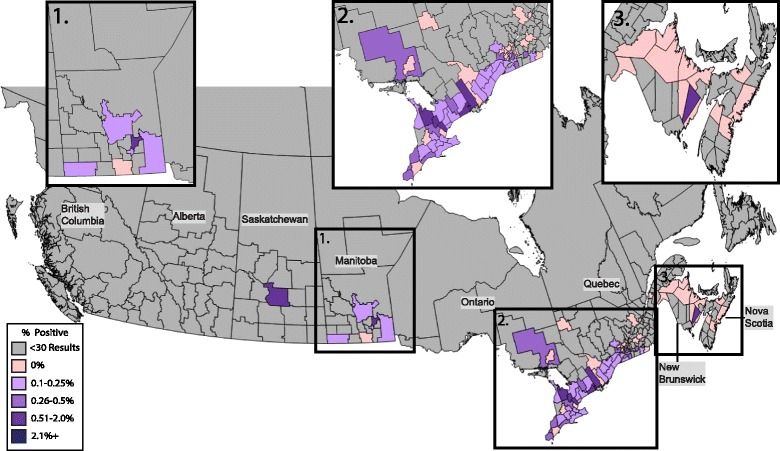
Percent positive antibody tests to *Ehrlichia* spp. in dogs by municipality. Evidence of antibody to *Ehrlichia* spp. in dogs by municipality throughout Canada, 2013–2014, grouped according to percent positive tests

Antibody to *Ehrlichia* spp. was identified in 0.19% of tests with a range among the provinces of 0–1.6% (Table 1). Saskatchewan had the highest seroprevalence of any province and was significantly higher than the national average (1.6%; *χ* ^2^ = 13.141, *P* = 0.0003). A total of 4 municipalities across Canada had a reported seroprevalence higher than 1%; Saskatoon, in central Saskatchewan (1.6%; 3/186; 95% CI: 0.33–4.9%), Hampton, in southern New Brunswick (1.3%; 2/152; 95% CI: 0.06–5.0%), and Bruce and Port Hope, in southwestern and southeastern Ontario, respectively (1.2%; 3/250; 95% CI: 0.24–3.6% and 1.0%; 6/590; 95% CI: 0.41–2.2%, respectively) (Fig. 4).

## Discussion

The dataset in the present paper was obtained from veterinarians in practice and allowed us to determine the prevalence of four vector-borne infections throughout Canada. As reported in previous studies, the data are biased towards major population centres where most dogs and dog owners reside [12]. While the prevalence of positive tests for heartworm antigen and antibody to *Ehrlichia* spp. and *Anaplasma* spp. were low in all provinces; there was evidence of past or current infection with at least one of these agents in every province reporting data (Table 1 and Figs. 1–4).

Percent positive tests for antibodies to *B. burgdorferi* were higher in the present study than reported in 2011 (0.72%; *P* < 0.0001), but not significantly different than more recent reports (2.1%; *P* = 0.70) [13, 14]. Moderate (> 1%) or high (> 5%) percent positive tests in dogs were identified in areas with frequent reports of human LB and where surveillance of ticks has confirmed the presence of *B. burgdorferi* [11, 39–41]. These areas are also near the northeastern or upper midwestern regions of the United States where LB is endemic or hyperendemic [12]. While the prevalence of *B. burgdorferi*-specific antibodies ranged from 0.5–15% for different provinces, there were also four municipalities with percent positive test results above 20%, the highest of which was Pictou County, in northern Nova Scotia at 40.6% (13/32). Areas such as Pictou County, southern Quebec, and eastern Ontario appear to constitute hyperendemic foci (> 5% positive tests) with a declining prevalence radiating outward (Fig. 1). This effect is likely exaggerated by human population clusters in southern Ontario but can also represent true foci of increased infection risk including the 11 municipalities in eastern Ontario where the seroprevalence is 5.1% *versus* the rest of the province with a seroprevalence of 0.87% (*P* < 0.0001).

Positive test results for heartworm antigen were most commonly seen near major population centres like Montreal and Toronto, with the rest of the municipalities reporting a prevalence of < 2% (Fig. 2). This urban-centered phenomenon is common in heartworm ecology in the US as domestic dogs serve as the major reservoir for infection of mosquitoes and large cities may harbour “heat islands” that create more favourable biologic conditions for the mosquitoes as compared to the surrounding rural areas [42]. While the total prevalence across Canada was quite low (0.42%) in the present paper, it was significantly higher than the previously described prevalence of 0.22% (*P* < 0.0001) [13]. Other studies have shown that heartworm prevalence in dogs in Canada has remained stable at approximately 0.2% over the last 30 years [21]. This apparent doubling in prevalence over the last five years may indicate increased testing of dogs in which infection is suspected, including dogs who have been adopted from areas where heartworm infections are endemic [43]. Alternatively, it could reflect a northward expansion of mosquito vectors due to changes in climate patterns in the region [44].

The analyte for *Anaplasma* spp. detects antibodies to both *A. phagocytophilum* and *A. platys. Anaplasma phagocytophilum* is transmitted by *I. scapularis*, like *B. burgdorferi*, and thus when mapped these two tick-borne infections often co-localize [12]. Some correlation between the two test results can be seen in this dataset, but it was not as strong as expected (Pearson’s correlation coefficient *ρ* = 0.34). While the municipalities with the highest *Anaplasma* spp. seroprevalence (> 2.0%) were associated with *B. burgdorferi* seroprevalence over 4.8% (*ρ* = 0.6), the municipalities with the highest prevalence of antibodies to *B. burgdorferi* (> 10%) did not correspond to high *Anaplasma* spp. seroprevalence (> 1%) (*ρ* = 0.17). *Anaplasma phagocytophilum* appears to circulate in nature at a lower level than *B. burgdorferi*, and detection of this pathogen in newly endemic areas may be difficult [4, 12, 39, 45]. The assays used in the present paper also detect antibody to *A. platys*, and it is not possible to differentiate that response from antibody to *A. phagocytophilum*. Reports of *R. sanguineus*, the vector for *A. platys*, are rare in Canada with less than 20 ticks reported per year in Ontario, in comparison to *I. scapularis,* which averages over 1,000 submissions each year [46]. Nonetheless, confirmed cases of *A. platys* in Canada have been reported as co-infections with *E. canis* and explained by travel to areas where *R. sanguineus* are more common [47].

Antibodies to *Ehrlichia* spp. were least commonly detected in the present study, likely due to a dearth of vector ticks in the region. As for *A. platys*, the risk for autochthonous transmission of *E. canis* by *R. sanguineus* in Canada is low, although travel cases may be diagnosed and reported [47]. Similarly, *A. americanum,* the vector of *E. ewingii* and *E. chaffeensis*, is still considered rare in this area of North America [29, 46]. Interestingly, the majority of positive tests for antibodies to *Ehrlichia* spp. were in southwestern Ontario, directly adjacent to the Midwest region of the United States that has now described *Ehrlichia muris-*like agent (EMLA) as a new *I. scapularis-*transmitted pathogen [48]. While more research is needed, existing data suggest antibodies to EMLA may be cross-reactive with existing assays for *Ehrlichia* spp. antibodies including that used in the present paper [38]. Although the natural maintenance cycle is not fully defined, EMLA has been identified in *I. scapularis* and white-footed mice (*Peromyscus leucopus)* [49, 50].

When nationwide data are collected, as in this study, there are limitations to the utility and interpretation of the data. Reporting bias, travel history, and detection method all factor into the prevalences presented [4]. In regions where low numbers of total tests are being reported, veterinarians may be using the SNAP® 4Dx® Plus Test Kit as a targeted diagnostic test rather than an annual wellness screening tool, a factor which may explain the high seroprevalence to *B. burgdorferi* reported from Nova Scotia (Table 1). Unfortunately, the current lack of data in western Canada prevents analysis in that region despite confirmation that *B. burgdorferi* is endemic in the northwestern United States and British Columbia [51]. It should also be noted that the low number of test results available in some areas and the low positive predictive values in low prevalence populations complicate interpretation [52, 53].

This nationwide data can aid veterinarians in making informed decisions on annual canine wellness procedures that would be most beneficial, including acaricide use, heartworm prevention, and vaccination for *B. burgdorferi*, and when evaluated over time, the results can help document the changing distribution of vector-borne infections [4, 12]. Finally, these vector-borne pathogens have been documented to cause disease in humans, and mapping the risk of canine infection also describes the areas where humans are most likely to be infected [32, 54, 55]. The species-specific nature of the *B. burgdorferi* analyte used in the SNAP® 4Dx® Plus Test kit may also allow for the differentiation of areas endemic for *B. burgdorferi* (*sensu stricto*) and those regions where other, or emerging, *Borrelia* spp. may be the main pathogen, allowing for more accurate diagnosis and specific treatments [30]. Further prevalence studies are warranted to investigate regions with no data at present and to provide updates on the changing distribution of these infections, particularly as they become newly endemic.

## Conclusions

This study serves as an update on the positive test results for common vector-borne infections in dogs, in Canada. Antibodies to *B. burgdorferi* were most commonly identified; the prevalence of infection in many provinces and the national average was higher than previously reported. While still low, percent positive *D. immitis* antigen tests were twice that reported 20 years ago, suggesting an increase in the prevalence of mosquito-borne heartworm. Infections with *Anaplasma* spp. and *Ehrlichia* spp. appear to remain fairly uncommon throughout Canada. While the work described here did not control for travel or false positives, canine serology may be a tool for monitoring vector-borne infections on a large scale and can be used to track the geographic spread of these agents and assess public health risks over time. Collectively, the data support efforts by veterinarians and physicians to protect pets and people from an increasing threat of vector-borne infections.

## Abbreviations

ELISA: Enzyme-linked immunosorbent assay
EMLA: *Ehrlichia muris*-like agent
IFA: Immunofluorescent assay
LB: Lyme borreliosis
WB: Western blot
